# Supplementary material for: Genotype Combinations Drive Variability in the Microbiome Configuration of the Rhizosphere of Maize/Bean Intercropping System
Source: Int J Mol Sci. 2024 Jan 20;25(2):1288. doi: 10.3390/ijms25021288 (PMC10815965; doi:10.3390/ijms25021288)
Supplement: Supplementary file 1 [file ijms-25-01288-s001.zip › ijms-2717719-supplementary.pdf]

Supplementary material

Table S1. Physical/chemical characteristics of the soil. The ratings are expressed according to the classifications published by the United States Department of Agriculture and the Italian Association of Public Agrochemical Laboratories. Soil analyses were performed by the Regional Agrochemical Centre Analysis Laboratory using standard operational procedures.

**Physiochemical properties of the soils**		**Ratings**
pH	8.22	alkaline
Soil texture		tending clayey
Sand (g/Kg)	178	
Silt (g/Kg)	425	
Clay (g/Kg)	397	
Active limestone (g/Kg)	126	very high
Total limestone (g/Kg)	234	moderately calcareous
Assimilable phosphorus (mg/Kg)	9.5	low
Exchangeable sodium (mg/Kg)	40	
Exchangeable calcium (mg/Kg)	5467	
Cation exchange capacity (meq/100g)	23.5	high
Assimilable iron (mg/Kg)	8.6	low
Assimilable manganese (mg/Kg)	3.3	very low
Assimilable zinc (mg/Kg)	0.43	low
Assimilable copper (mg/Kg)	3.4	medium
Soluble boron (mg/Kg)	0.25	low
C/N	9.3	regular
Organic matter		low
Total nitrogen	1.05	medium
Mg/K	6.1	
Exchangeable magnesium (mg/Kg)	389	high
Exchangeable phosphorus (mg/Kg)	204	high

Table S2. Anova on Alpha-diversity indexes for Plant status and Cropping system. Results of Anova applied to Chao1 and Shannon index values to test the effect of Cropping System and Plant Status.

**Chao1**	Df	Sum Sq	Mean Sq	F value	Pr(>F)
Year	1	178667	178667	0.549	0.461
Replicates	2	299654	149827	0.46	0.633
Cropping system	1	134212	134212	0.412	0.523
Plant status	1	1349759	1349759	4.144	0.045*
Residuals	89	28988579	325714		
**Shannon**	Df	Sum Sq	Mean Sq	F value	Pr(>F)
Year	1	0.07	0.069	1.666	0.200
Replicates	2	0.089	0.045	1.072	0.347
Cropping system	1	0.009	0.009	0.216	0.643
Plant status	1	0.177	0.177	4.245	0.042*
Residuals	89	3.715	0.042		

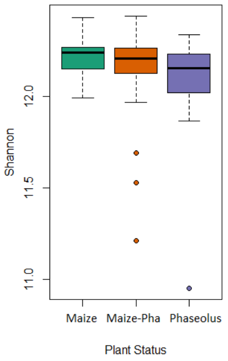

Figure S1. Means of Shannon diversity index for Plant Status. Boxplots of Shannon diversity index values for means of maize and beans in SC and for the IC condition (Plant Status).

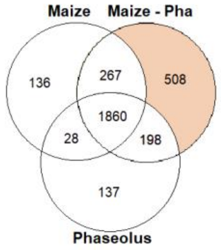

Group	Counts	Abundance
Maize	136	1973
Maize - Pha	508	10334
Phaseolus	137	1684
Maize&Maize-Pha	267	22702
Maize&Phaseolus	28	1579
Phaseolus&Maize-Pha	198	9912
Maize&Maize-Pha&Phaseolus	1860	41921105

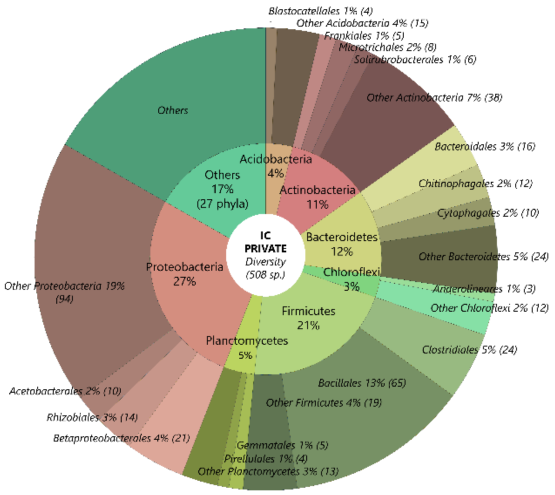

(**a**) (**b**)

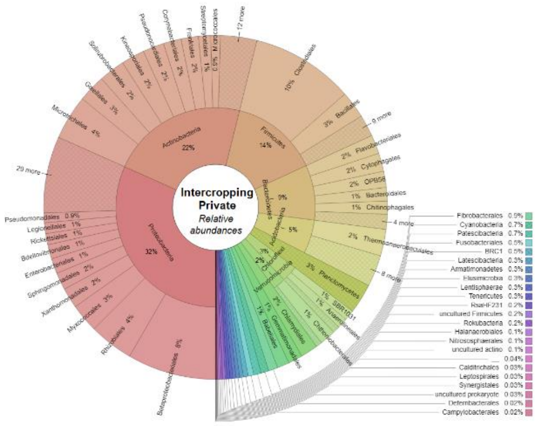

(c)

Figure S2. Diversity and composition of private bacterial members of IC condition. (A) Venn diagram of bacterial species belonging to each subset with number of taxa and total reads for each subset. (B) Doughnut plot of total bacterial diversity for private members of IC condition with percentages of phyla in the inner circle and orders in the outer circle. (C) Krona plot of relative abundances of the bacterial members that are private of the IC condition.

Table S3. Multi-factorial Anova on Chao1 index to test the effect of genotypes combinations in IC. Results of the multi-factorial Anova applied to Chao1 diversity index values.

	Df	Sum Sq	Mean Sq	F value	Pr(>F)
Year	1	22171	22171	0.069	0.793
Replicates	2	771458	385729	1.202	0.305
Environment	4	1461652	365413	1.138	0.343
Replicates:Environment	8	3101719	387715	1.208	0.303
Environment:Accession	23	14361557	624416	1.945	0.013 *
Year:Replicates	2	752579	376290	1.172	0.314
Year:Replicates:Environment	12	5332940	444412	1.384	0.187
Year:Replicates:Environment:Accession	49	14752634	301074	0.938	0.591
Residuals	95	30495176	321002		

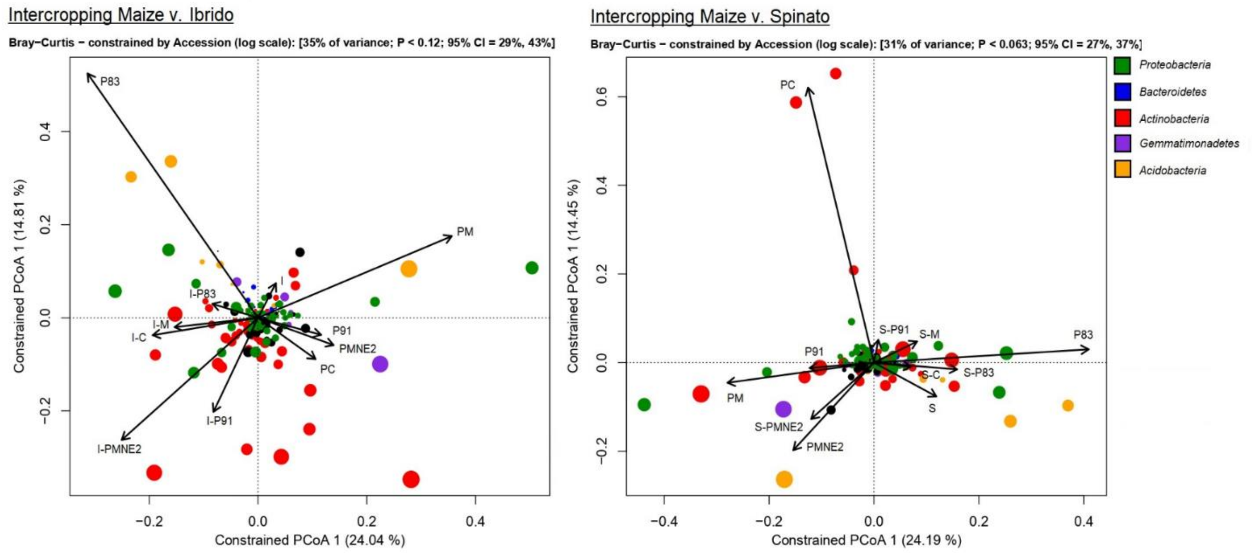

Figure S3. CPcoA for IC combinations with maize I and S. Constrained Principal Coordinate analysis (PcoA) and biplot of phyla scores of PcoA analysis based on Bray-Curtis distances of rhizosphere samples IC systems of S and I, constrained by "Accessions" including maize and beans in SC and different IC combination with the maize line. The percentage of variation explained by each axis refers to the fraction of the total variance of the data explained by the constrained factor (35% and 31% for I and S systems, respectively). CAP analysis failed to show a significant effect on sample clustering by "Accessions" (p < 0.12 and p < 0.063 in I and S, respectively). The arrows point to the centroid of the constrained factor.

Circle size depicts the relative abundance of phyla (log scale) that were more contributing in clustering samples. Colors illustrate different phyla as reported in the legend.

Table S4. p-values and LDA scores of LefSE analysis. Table showing results derived by the Linear discriminant analysis Effect Size (LEfSe) for the bacterial communities at genus level identifying biomarkers between Maize and Phaseolus in SC and Maize – Phaseolus in IC.

Taxa	Group	pvalue	LDA
k__Bacteria|p__Proteobacteria|c__Gammaproteobacteria|o__Betaproteobacteriales	Maize	0.04011	3.986829
k__Bacteria|p__Proteobacteria|c__Gammaproteobacteria|o__Betaproteobacteriales|f__Burkholderiaceae	Maize	0.04692	3.946383
k__Bacteria|p__Proteobacteria|c__Gammaproteobacteria|o__Betaproteobacteriales|f__Nitrosomonadaceae	Phaseolus	0.00342	3.891149
k__Bacteria|p__Proteobacteria|c__Gammaproteobacteria|o__Betaproteobacteriales|f__Nitrosomonadaceae|g__MND1	Phaseolus	0.00484	3.868153
k__Bacteria|p__Actinobacteria|c__Actinobacteria	Maize-Pha	0.01247	3.839024
k__Bacteria|p__Actinobacteria|c__Actinobacteria|o__Propionibacteriales|f__Nocardioidaceae|g__Nocardioides	Maize-Pha	0.00349	3.528047
k__Bacteria|p__Actinobacteria|c__Actinobacteria|o__Propionibacteriales	Maize-Pha	0.00615	3.495445
k__Bacteria|p__Actinobacteria|c__Actinobacteria|o__Propionibacteriales|f__Nocardioidaceae	Maize-Pha	0.00615	3.495445
k__Bacteria|p__Actinobacteria|c__Actinobacteria|o__Streptomycetales	Maize-Pha	0.02654	3.486422
k__Bacteria|p__Actinobacteria|c__Actinobacteria|o__Streptomycetales|f__Streptomycetaceae	Maize-Pha	0.02654	3.486422
k__Bacteria|p__Actinobacteria|c__Actinobacteria|o__Streptomycetales|f__Streptomycetaceae|g__Streptomyces	Maize-Pha	0.02654	3.486422
k__Bacteria|p__Proteobacteria|c__Deltaproteobacteria|o__Myxococcales|f__Haliangiaceae	Phaseolus	0.04737	3.472508
k__Bacteria|p__Proteobacteria|c__Deltaproteobacteria|o__Myxococcales|f__Haliangiaceae|g__Haliangium	Phaseolus	0.04737	3.472508
k__Bacteria|p__Actinobacteria|c__Actinobacteria|o__Micrococcales	Maize	0.01431	3.411162
k__Bacteria|p__Verrucomicrobia	Maize	0.02435	3.293216
k__Bacteria|p__Verrucomicrobia|c__Verrucomicrobiae	Maize	0.02435	3.293216
k__Bacteria|p__Verrucomicrobia|c__Verrucomicrobiae|o__Pedosphaerales	Maize	0.02332	3.199208
k__Bacteria|p__Verrucomicrobia|c__Verrucomicrobiae|o__Pedosphaerales|f__Pedosphaeraceae	Maize	0.02332	3.199208
k__Bacteria|p__Proteobacteria|c__Deltaproteobacteria|o__NB1-j	Phaseolus	0.02816	3.190599
k__Bacteria|p__Actinobacteria|c__Actinobacteria|o__Pseudonocardiales|f__Pseudonocardiaceae|g__Lechevalieria	Maize	0.01147	3.162907
k__Bacteria|p__Actinobacteria|c__Actinobacteria|o__Micrococcales|f__Micrococcaceae	Maize	0.00929	3.072155
k__Bacteria|p__Entotheonellaeota	Maize-Pha	0.01402	3.058515
k__Bacteria|p__Entotheonellaeota|c__Entotheonellia	Maize-Pha	0.01402	3.058515
k__Bacteria|p__Entotheonellaeota|c__Entotheonellia|o__Entotheonellales|f__Entotheonellaceae	Maize-Pha	0.01402	3.058515
k__Bacteria|p__Entotheonellaeota|c__Entotheonellia|o__Entotheonellales	Maize-Pha	0.01402	3.058515
k__Bacteria|p__Actinobacteria|c__Actinobacteria|o__Micrococcales|f__Microbacteriaceae|g__Agromyces	Maize-Pha	0.02815	3.017008
k__Bacteria|p__Actinobacteria|c__Actinobacteria|o__Micrococcales|f__Microbacteriaceae	Maize-Pha	0.02815	3.017008
k__Bacteria|p__Proteobacteria|c__Gammaproteobacteria|o__Pseudomonadales	Maize	0.02981	3.016641
k__Bacteria|p__Proteobacteria|c__Gammaproteobacteria|o__Pseudomonadales|f__Pseudomonadaceae|g__Pseudomonas	Maize	0.02981	3.016641
k__Bacteria|p__Proteobacteria|c__Gammaproteobacteria|o__Pseudomonadales|f__Pseudomonadaceae	Maize	0.02981	3.016641
k__Bacteria|p__Actinobacteria|c__Actinobacteria|o__Corynebacteriales	Maize-Pha	0.04192	2.963743
k__Bacteria|p__Verrucomicrobia|c__Verrucomicrobiae|o__Verrucomicrobiales|f__Rubritaleaceae	Maize	0.00047	2.93693
k__Bacteria|p__Verrucomicrobia|c__Verrucomicrobiae|o__Verrucomicrobiales	Maize	0.00047	2.93693
k__Bacteria|p__Verrucomicrobia|c__Verrucomicrobiae|o__Verrucomicrobiales|f__Rubritaleaceae|g__Luteolibacter	Maize	0.00047	2.93693
k__Bacteria|p__Proteobacteria|c__Deltaproteobacteria|o__Myxococcales|f__Archangiaceae	Maize-Pha	0.04081	2.734768
k__Bacteria|p__Actinobacteria|c__Actinobacteria|o__Micrococcales|f__Intrasporangiaceae	Maize-Pha	0.01	2.733414
k__Bacteria|p__Cyanobacteria|c__Oxyphotobacteria|o__Chloroplast|f__Phaseolus acutifolius (tepary bean)	Phaseolus	0.01608	2.604061
k__Bacteria|p__Cyanobacteria|c__Oxyphotobacteria|o__Chloroplast|f__Phaseolus acutifolius (tepary bean)|g__Phaseolus acutifolius (tepary bean)	Phaseolus	0.01608	2.604061
k__Bacteria|p__Cyanobacteria|c__Oxyphotobacteria|o__Chloroplast|f__Phaseolus acutifolius (tepary bean)|g__Phaseolus acutifolius (tepary bean)|s__Phaseolus acutifolius (tepary bean)	Phaseolus	0.01608	2.604061

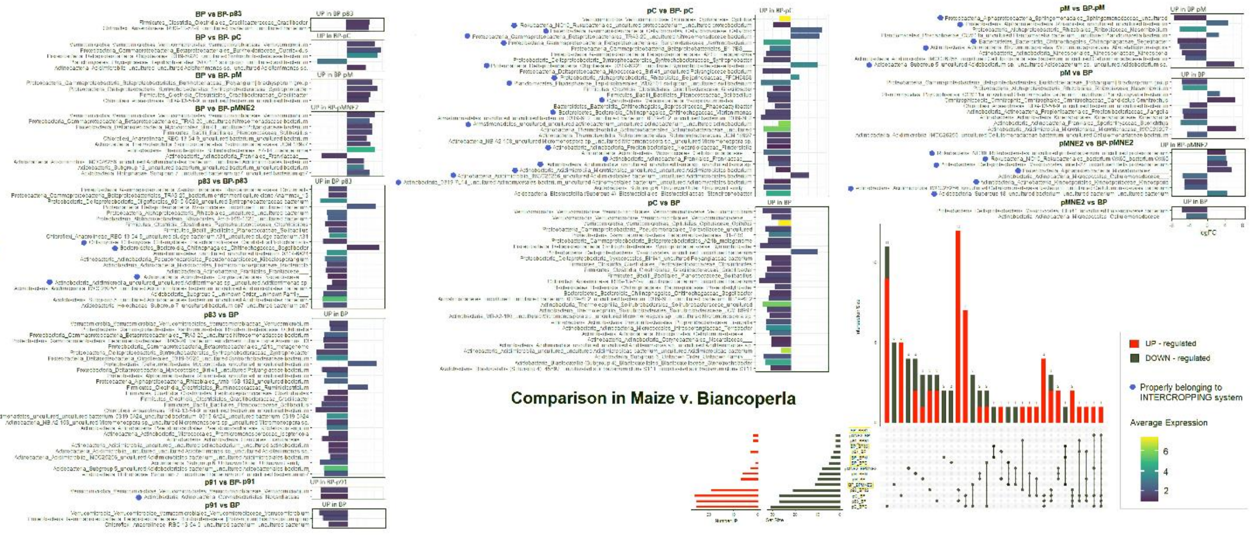
Comparison in Maize v. Biancoperla
UP - regulated
DOWN - regulated
Properly belonging to INTERCROPPING system
Average Expression

(a)

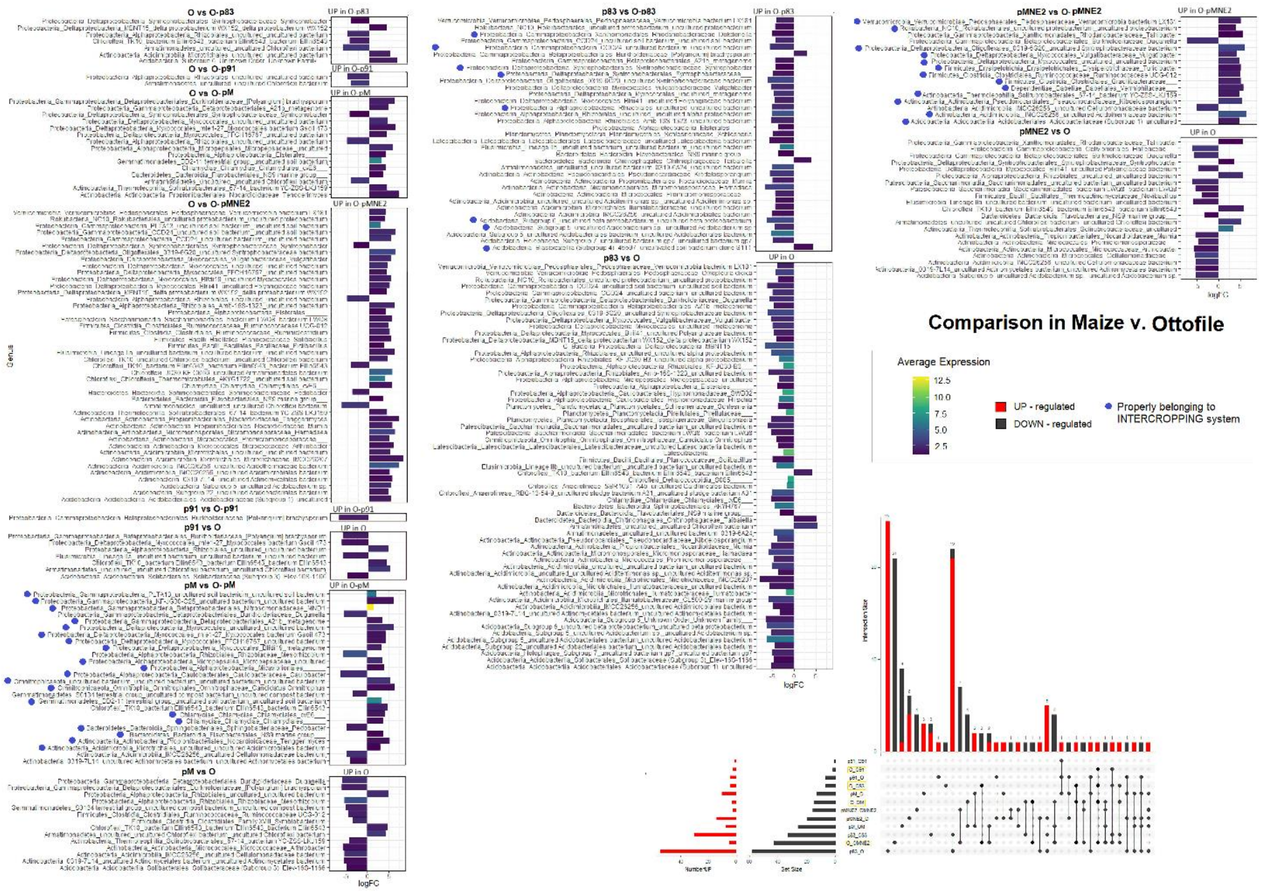
Comparison in Maize v. Ottofile
Average Expression
UP - regulated
DOWN - regulated
Properly belonging to INTERCROPPING system

(b)

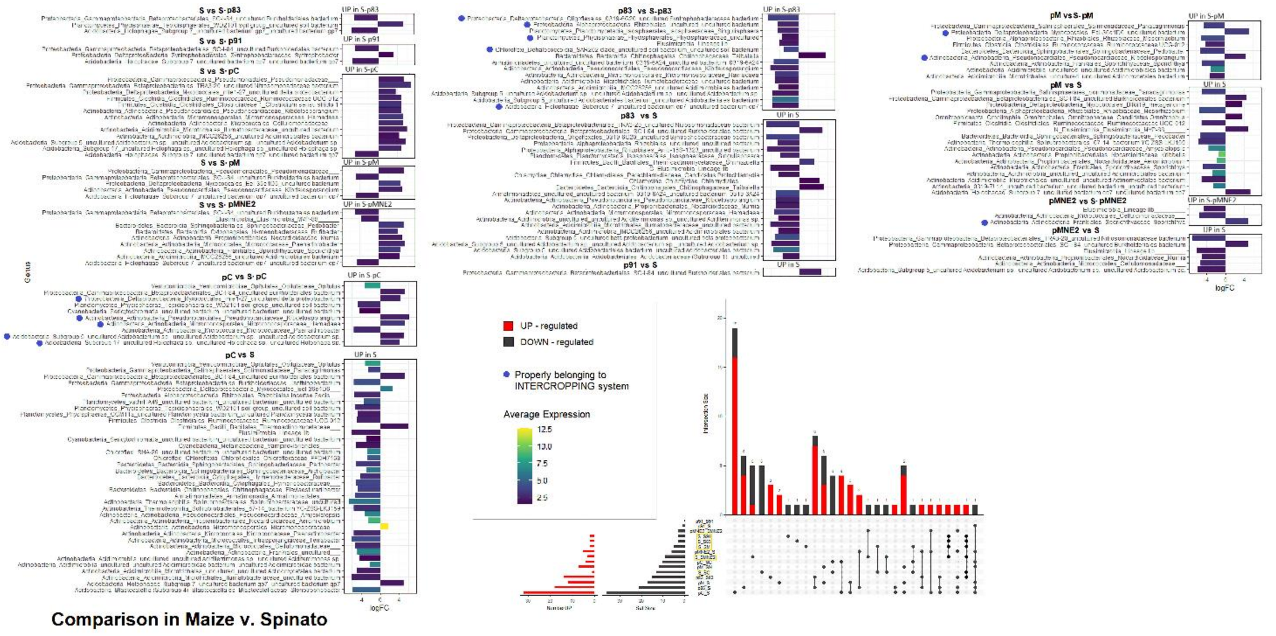

(c)

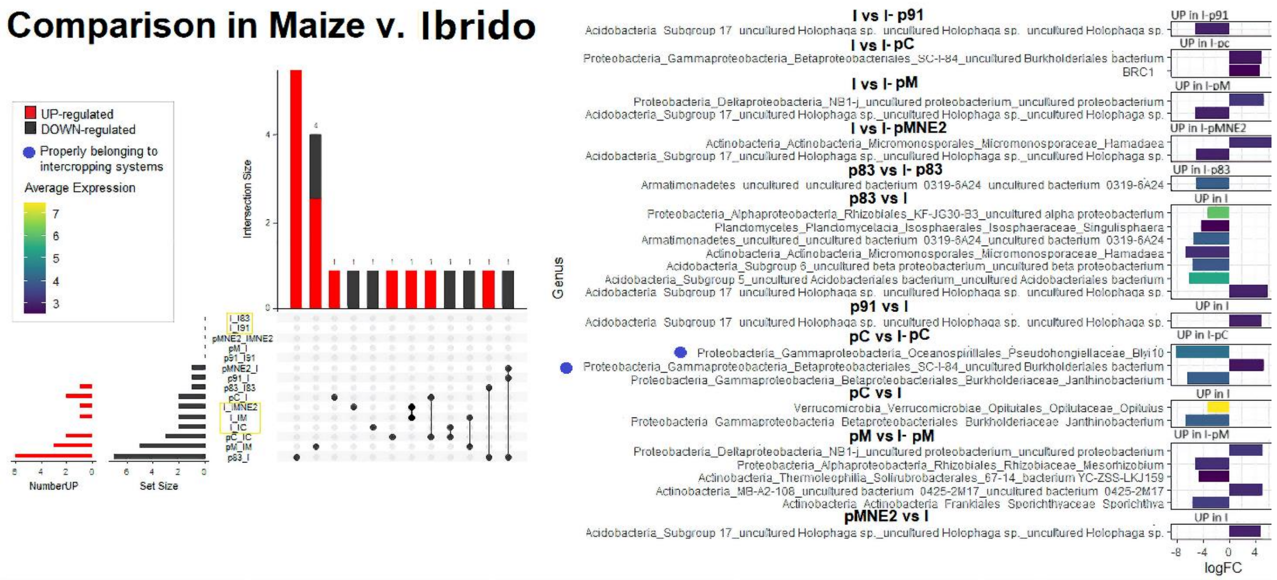

(d)

Figure S4. Bacterial genera differentially expressed in IC combinations. Log2 Fold Changes and average expressions of bacterial genera differentially expressed obtained with the FitZig model applied at "Accession" level on log transformed counts for each "Environment" (A. Biancoperla, B. Ottofile, C. Spinato, D. Ibrido). Bacterial genera are grouped by phyla and all genera differentially expressed are reported. Intersection plots showing genera differentially expressed and up and down regulated in each comparison are reported. Blue dots are reported in correspondence of bacterial genera that properly belong to the intercropping system.

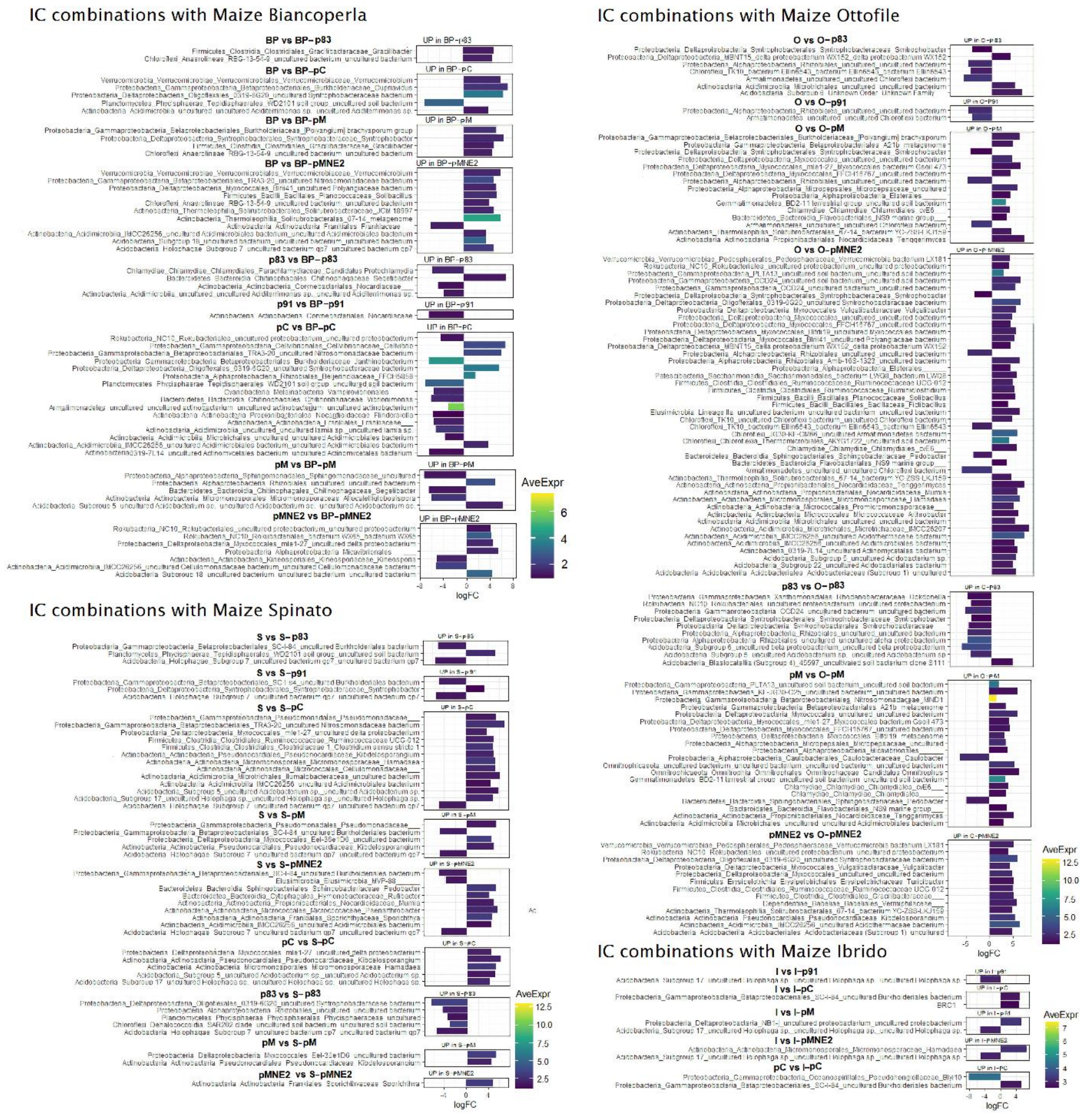

Figure S5. Bacterial genera differentially expressed in IC combinations and proper of IC condition. Log2 Fold Changes and average expressions of bacterial genera differentially expressed obtained with the FitZig model applied at "Accession" level on log transformed counts for each "Environment". Bacterial genera are grouped by phyla and only bacterial genera proper of the IC condition are reported (taxa DE between beans and maize in SC and in common with taxa DE in beans in SC versus IC were excluded).
